# Long-Read Metagenomics Reveals Temporal DNA Virome Dynamics and Recovers Sequences of a Major Viral Pathogen at a Sea-Cage Fish Farm

**DOI:** 10.3390/microorganisms14102362

**Published:** 2026-10-15

**Authors:** Ahran Kim, Sung-Bin Moon, Ju-sung Kim, Myoung Sug Kim, Yoonhang Lee, Gyoungsik Kang, Kyung-Ho Kim, HyeongJin Roh

**Affiliations:** 1 Pathology Research Division, National Institute of Fisheries Science (NIFS), Busan 46083, Republic of Korea; 2 Department of Aquatic Life Medicine, College of Marine Sciences, Gyeongsang National University, Tongyeong 53064, Republic of Korea; 3 Department of Aqualife Medicine, Chonnam National University, Yeosu 59626, Republic of Korea

**Keywords:** virome, RSIV, *Megalocytivirus*, seawater, aquaculture, virus–host coupling, nanopore sequencing

## Abstract

Coastal sea-cage aquaculture environments support dynamic microbial communities, yet their viral components remain poorly characterized. This study profiled DNA viral-like reads recovered from total metagenomes of iron-flocculated seawater concentrates (operationally termed DNA virome) at a rock bream (*Oplegnathus fasciatus*) sea-cage farm off Geoje Island, Korea, at five time points between July 2024 and May 2025 using long-read Nanopore metagenomics. Multivariate analyses of DNA virome composition indicated temporal patterning in virome composition; samples collected within the cages tended to group together. The DNA virome was dominated overall by bacteriophages of abundant marine bacteria and by viruses infecting picoeukaryotic phytoplankton, and it showed marked temporal turnover during the investigated year: *Micromonas*-associated viruses became prominent in December, whereas *Ostreococcus*- and *Bathycoccus*-associated viruses increased during seasonal rewarming in May, suggesting that environmentally driven changes in microbial host communities may contribute to temporal virome turnover. *Pelagibacter* phage abundance was positively correlated with that of its potential host *Pelagibacter ubique*, and both *Synechococcus* T7-like virus and *Synechococcus* virus were positively correlated with *Parasynechococcus marenigrum*, associations that are consistent with, but do not demonstrate, taxon-specific virus–host relationships. Nanopore reads assigned to infectious spleen and kidney necrosis virus, now classified within the species *Megalocytivirus pagrus 1*, were recovered from samples collected in September 2024 (12,960 bp) and May 2025 (340 bp). Average nucleotide identity analysis indicated that the seawater-derived sequence was most closely related to red seabream iridovirus subtype II, the subtype predominant in Korean cultured marine fish. The seawater virome results and the bioinformatic pipelines developed in this study together may provide a non-invasive approach for tracking temporal virome dynamics and for environmental screening of viral pathogen DNA in sea-cage aquaculture.

## 1. Introduction

Coastal marine aquaculture environments are shaped by multiple environmental factors, including water temperature, nutrient availability, hydrodynamic conditions, and the organic inputs associated with farming activities [1,2,3], and these factors can reshape the composition and ecological functions of the surrounding microbial communities [4]. A previous study conducted at rock bream (*Oplegnathus fasciatus*) sea-cage farms off Geoje Island, Korea, accordingly revealed pronounced seasonal shifts in the seawater microbiome that coincided with changes in environmental conditions [5]. That study also demonstrated the utility of long-read metagenomic sequencing for resolving temporal microbial dynamics and for detecting potential bacterial pathogen exposure signals and antimicrobial resistance genes in aquaculture waters.

Changes in microbial communities are likely to influence the viruses that infect them. Marine viruses are abundant and genetically diverse components of aquatic ecosystems, and their replication and population dynamics are closely tied to the availability and physiological state of their microbial hosts [6,7]. Through host lysis, viruses regulate microbial population turnover, redirect organic matter and nutrients through the viral shunt, and contribute to horizontal gene transfer and microbial evolution [8,9]. Bacteriophages infecting marine bacteria and cyanobacteria, together with viruses infecting picoeukaryotic phytoplankton, may therefore track seasonal changes in ecologically important microbial populations. Despite this ecological importance, viral communities in sea-cage aquaculture environments remain considerably less studied than bacterial communities, and the relationships between seawater microbiome and virome dynamics in these systems are still poorly understood.

Aquaculture production is also threatened by diverse viral pathogens capable of causing acute disease outbreaks and substantial economic losses [10]. In Korean marine aquaculture, megalocytiviruses are among the most important of these pathogens. Red seabream iridovirus (RSIV), a genotype within the species *Megalocytivirus pagrus 1*, causes systemic infection and high mortality in cultured marine fish, including rock bream, and outbreaks typically occur during the warmer months. Genome-based analyses have further divided RSIV into subtypes I, II and A, with subtype II predominating in Korean cultured fish [11,12,13]. Conventional surveillance generally relies on pathogen-specific molecular assays, which require the target pathogen to be selected in advance and frequently entail invasive or lethal sampling of cultured fish [3]. Untargeted virome analysis, by contrast, can characterize the broader viral community and recover pathogen-derived sequences directly from environmental samples in a single workflow [14,15,16]. Long-read sequencing platforms such as Oxford Nanopore provide extended sequence information that can improve viral taxonomic assignment and comparison among closely related viral lineages [17,18], and where sufficient informative genomic regions are recovered, these sequences may also identify the viral group or genotype most closely related to the environmental signal. Bioinformatic pipelines for seawater virome analysis could therefore offer a non-invasive route to broad viral pathogen screening and subsequent genetic characterization. Such pipelines nevertheless remain insufficiently optimized for aquaculture environments, which limits the application of seawater virome data to environmental pathogen surveillance.

In the present study, we characterized virus-associated DNA sequences (hereafter, the DNA virome) recovered from the same seawater samples examined in the companion microbiome study [5]. We aimed specifically to (i) characterize seasonal and site-associated changes in seawater viral community composition, (ii) identify dominant viral taxa and evaluate abundance relationships between selected bacteriophages and their potential prokaryotic hosts, and (iii) determine whether untargeted long-read metagenomics could detect sequences related to aquaculture-associated viral pathogens and provide sufficient genomic information for their preliminary sequence-based classification. This integrated analysis was designed to evaluate the ecological significance of seawater virome dynamics and the potential utility of virome monitoring for detecting environmental signals of viral pathogen exposure in sea-cage aquaculture.

## 2. Materials and Methods

### 2.1. Seawater Sampling Information

**Figure 1:**
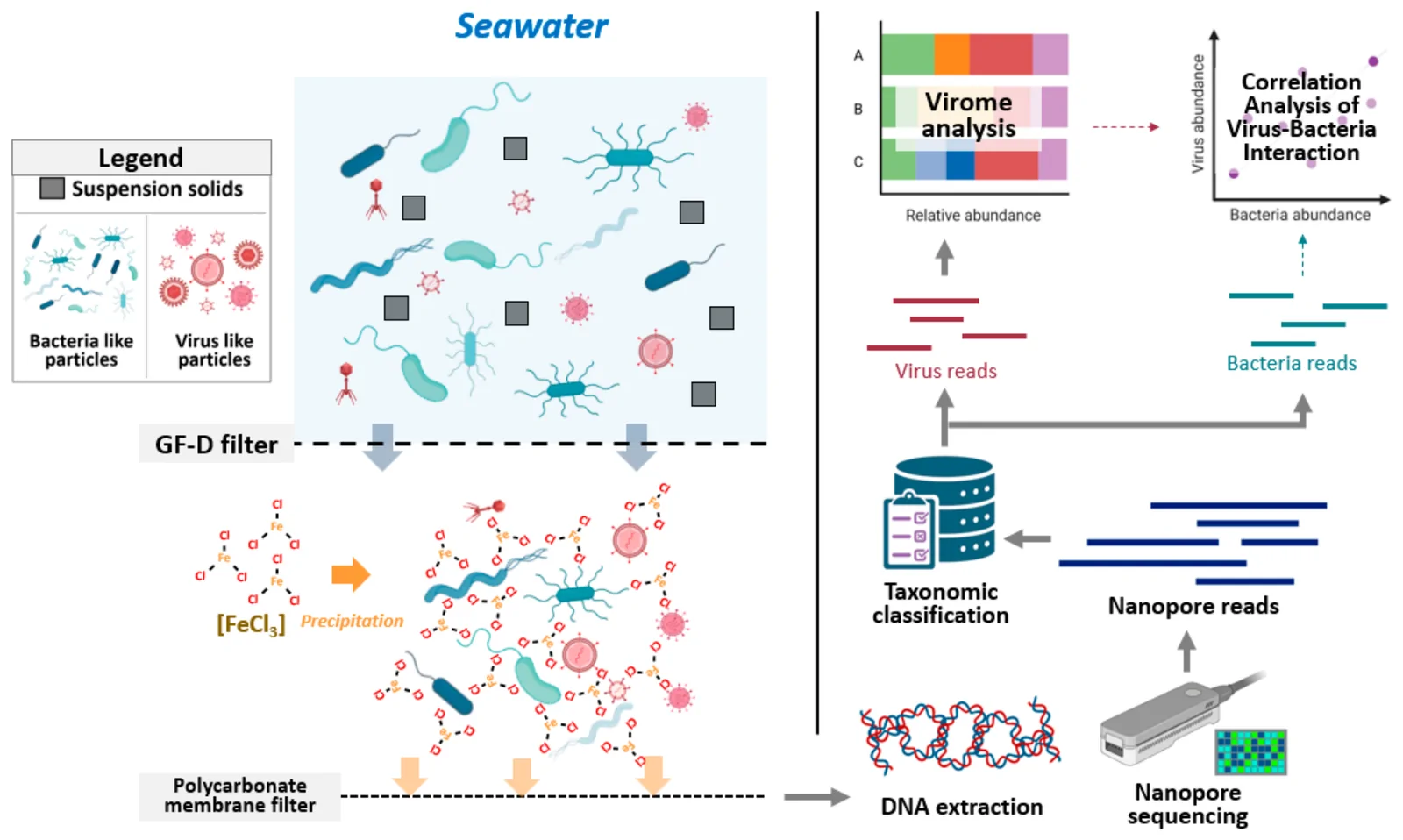
Schematic overview of the workflow for seawater virome analysis and virus–bacteria interaction analysis. Seawater samples were pre-filtered through a GF/D filter, followed by FeCl_3_-mediated precipitation and collection on a polycarbonate membrane filter. Extracted DNA was subjected to Nanopore sequencing and taxonomic classification, followed by virome profiling and correlation analysis between viral and bacterial abundances to explore potential virus–bacteria interactions among samples.

Seawater samples were collected from a rock bream (*Oplegnathus fasciatus*) sea-cage farming area in Geoje, Korea, at two to three locations per sampling occasion (Figure 1) [5]. Sites V and NV were located inside the cages of two adjacent farms, and samples were taken from the rearing water within the cages. Site M was located in the passage between the two cage groups, and site O200m was located outside the farming area, approximately 200 m from the cages. Sampling was carried out at five time points across different seasons: July 2024 (2024_July; V_2407, NV_2407, and M_2407), September 2024 (2024_Sep; V_2409 and NV_2409), December 2024 (2024_Dec; V_2412, NV_2412, and O200m_2412), February 2025 (2025_Feb; V_2502, NV_2502, and O200m_2502), and May 2025 (2025_May; V_2505, NV_2505, and O200m_2505). The sites sampled were V, NV, and M in July 2024; V and NV in September 2024; and V, NV, and O200m in December 2024, February 2025, and May 2025, following the sampling design described previously [5]. Mean seawater temperatures in the waters around Geoje Island were 19.6 ± 1.0 °C in July 2024, 27.4 ± 1.3 °C in September 2024, 14.6 ± 0.9 °C in December 2024, 10.7 ± 0.4 °C in February 2025, and 15.0 ± 1.1 °C in May 2025. One 500 mL seawater sample was collected per site at each time point, giving 14 samples that were processed and sequenced individually. Samples were collected in the field and transported promptly to the laboratory for further analysis.

### 2.2. Concentration of Bacteria and Viruses in Seawater

To capture both bacterial and viral particles, the iron flocculation method of a previous study was modified [19]. Briefly, 500 mL of each seawater sample was first passed through a GF/D glass microfiber filter (pore size: 2.7 μm) to remove suspended solids, eukaryotes, and plankton while allowing most bacterial and virus-like particles to pass through. The resulting filtrate was then subjected to iron flocculation for the simultaneous concentration of bacterial and viral particles, as described in Kim et al. [5]. Iron flocculates were collected on a 0.8 μm polycarbonate filter, eluted with EDTA–MgCl_2_–oxalic acid buffer at 4 °C for 24 h, and recovered in a final volume of approximately 1 mL. As the protocol was intended to broadly capture DNA associated with both bacterial and viral communities, sub-0.22 μm size fractionation was not applied. Total DNA was then extracted using the AccuPrep^®^ DNA Extraction Kit (Bioneer, Daejeon, Republic of Korea) according to the manufacturer’s instructions (Figure 1). No DNase treatment, density-gradient purification, or other viral-particle purification was applied to the samples before DNA extraction. Accordingly, the term “DNA virome” is used operationally in this study to denote the virus-associated sequences identified within this total metagenomic DNA, rather than a sequencing library consisting exclusively of encapsidated viral genomes.

### 2.3. Nanopore Sequencing and Bioinformatics

Raw Nanopore sequencing data generated in an earlier study [5] were obtained and used for the present analysis. The sequencing libraries had been prepared using the Native Barcoding Kit 24 V14 (Oxford Nanopore Technologies, Oxford, UK) and sequenced on the Oxford Nanopore platform using a MinION device with an R10.4.1 flow cell; accession numbers for the raw sequencing data are provided in Table A1. Reads with a mean Phred quality score above 10 were retained during basecalling using MinKNOW software (Ver. 25.09.16). The taxonomic composition of the non-viral fraction of the same sequencing data, which was dominated by bacteria, has been shown in a former study [5]. To identify virus-like reads, all sequence reads were searched against the NCBI Viral Reference Database using BLASTn (Ver. 2.12.0+). Reads were classified as viral when the best-matching reference viral sequence met the following criteria: E-value < 1 × 10^−5^, query coverage ≥ 70%, and percent identity ≥ 80%. As only viral reference sequences were included in the database, a relatively high query coverage threshold was applied so that assignments were based on alignment across most of each long read rather than on short regions that may be shared with cellular genomes. The total sequencing output of each sample, together with the amount and proportion of bases assigned to viral reference sequences at E-value < 1 × 10^−5^ alone and under the full set of thresholds, is summarized in Table A2. Reads satisfying these criteria were retained as virus-like reads for subsequent analyses, and the abundance of each viral species was expressed as a normalized viral coverage length ratio, defined in the equation below as the percentage of aligned bases assigned to that species relative to the total aligned bases of all virus-like reads in the same sample. Taxonomic classification was based on the best-matching reference sequence of each read that met the above criteria, and the results were aggregated at the species level according to the species of that reference sequence, after which the abundance profiles of the 10 most abundant viral species were examined across seawater samples. To characterize the overall variation in virome composition, the species-level normalized viral coverage length ratios were used for three exploratory analyses. Three-dimensional principal component analysis (3D PCA) was performed with prcomp on the centered, unscaled ratios. NMDS was performed with metaMDS in vegan (k = 2), which used Bray–Curtis dissimilarity and, under its default settings, applied a square-root transformation followed by Wisconsin double standardization to down-weight the most abundant taxa in these compositional data. Hierarchical clustering used Ward.D linkage on Euclidean distances.

$$Normalized\;viral\;coverage\;length\;ratios=\;\frac{Total\;bases\;assigned\;to\;each\;viral\;species\;per\;sample}{Total\;bases\;of\;viral\;reads\;passing\;the\;filtering\;thresholds\;per\;sample}\times 100$$

Because this ratio is calculated within each sample, it expresses the relative contribution of each viral species to the virus-like fraction and does not depend directly on the total sequencing yield of that sample, which differed among samples (Table A2). It does not, however, represent absolute viral abundance, and its precision decreases as the number of virus-like bases in a sample decreases. The total number of virus-like bases underlying each profile is therefore reported in Table A2.

### 2.4. Correlation Between Major Viruses and Their Potential Bacterial Hosts

Among the 10 most abundant viral taxa, *Pelagibacter* phage and *Synechococcus* phage were selected as the major bacteriophages, because their abundances varied pronouncedly with season and sampling site. The normalized prokaryotic coverage length ratios reported previously by Kim et al. [5] were used as measures of bacterial abundance in the correlation analyses. Spearman’s rank correlation analyses were then performed across all 14 samples (*n* = 14 for each correlation) to evaluate the relationship between the abundance of each phage and that of its potential bacterial host taxa, *Pelagibacter* spp. and *Synechococcus* spp., respectively. To account for multiple testing, *p*-values were adjusted using the Benjamini–Hochberg false-discovery-rate procedure within each set of correlations using R. Associations with adjusted *p*-values (q-value) < 0.05 were regarded as statistically supported.

### 2.5. Genotyping of the Seawater-Derived Megalocytivirus

To genotype the pathogenic iridovirus detected in the seawater samples, raw Nanopore reads were mapped against the RSIV reference genome (17RbGs strain; OK042109.1) using minimap2 (ver. 2.28) [20]. Reads mapped to the RSIV genome were then extracted with samtools (ver. 1.9) and bedtools (ver. 2.31.1) for subsequent genotyping analysis [21,22]. Within each of these reads, the nucleotide regions aligning to RSIV in the BLASTn output were used to define the coordinates for the subsequent analysis [23]. The alignment coordinates of the confirmed reads on the major RSIV genomes were then identified, and the homologous regions corresponding to these coordinates were extracted from the reference genomes of major RSIV strains (RSIV_121, MT798582.1; RIE12-1, AP017456.1; 17RbGs, OK042109.1; KagYT-96, MK689686.1; OSGIV, AY894343.1; RBIV_C1, KC244182.1; RBIV_KOR_TY1, AY532606.1; 17SbTy, OK042108.1; Ehime-1, AB104413.1; SBIV-V12, ON743042.1; and SBIV-VP13, ON740976.1) and of other closely related iridoviruses (ISKNV, AF371960.1 and KT781098.1; TRBIV, GQ273492.1). For each strain, the extracted regions were arranged in a consistent genomic order and concatenated, using only the reads recovered from the September sample; the short sequence detected in May was not included, after which pairwise average nucleotide identity (ANI) was calculated among the resulting sequences using FastANI [24]. Hierarchical clustering based on the ANI matrix was finally performed to determine the genetic relationship between the seawater-derived iridovirus sequence and the reference iridovirus strains.

### 2.6. Data Visualization and Statistical Analysis

Statistical analyses and data visualization were performed in R (version 4.3.1) using the “vegan”, “rgl”, “factoextra”, “ape”, “phyloseq”, “tidyverse”, “ggplot2”, “corrplot”, “ggcorrplot”, “ellipse”, and “pheatmap” packages. Differences in virome composition among sampling dates were tested by PERMANOVA (adonis2, 999 permutations) on the same Bray–Curtis dissimilarities used for NMDS, homogeneity of multivariate dispersion was assessed with betadisper (999 permutations), and seawater temperature was fitted to the NMDS ordination using envfit (999 permutations) and tested by PERMANOVA as a continuous variable. Correlations were evaluated by Spearman’s rank correlation analysis, and q-values < 0.05 were considered statistically significant.

## 3. Results and Discussion

### 3.1. Temporal and Spatial Dynamics of the Seawater Virome

**Figure 2:**
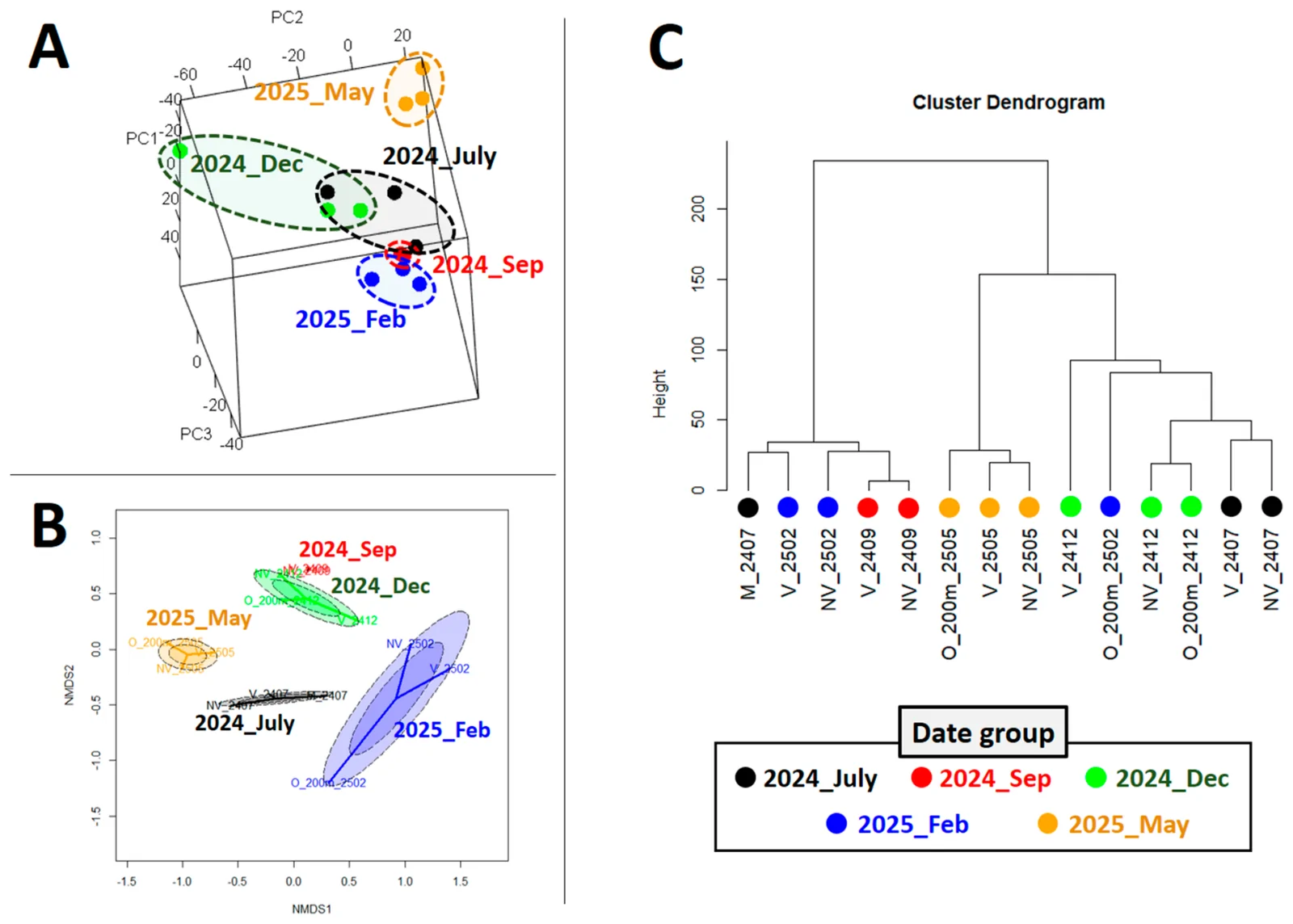
Ordination and hierarchical clustering analyses of temporal variation in seawater virome composition. (**A**) Three-dimensional principal component analysis (PCA) plot, showing the first three principal components (PC1: 55.1%, PC2: 22.9%, and PC3: 12.1% of the total variance). Points represent samples grouped by collection date: July 2024 (2024_July, black), September 2024 (2024_Sep, red), December 2024 (2024_Dec, green), February 2025 (2025_Feb, blue), and May 2025 (2025_May, orange). (**B**) Non-metric multidimensional scaling (NMDS) plot based on Bray–Curtis dissimilarities of normalized viral coverage length ratios (metaMDS, k = 2; stress = 0.109). Distances between points indicate similarities in virome composition, and colors correspond to the sampling dates described above. Ellipses denote confidence limits of 0.25 (dark shading) and 0.5 (light shading) for each group. (**C**) Hierarchical clustering dendrogram constructed using Ward.D linkage on Euclidean distances between samples, calculated from normalized viral coverage length ratios at the viral species level.

Three-dimensional PCA, in which the first three components together explained 90.1% of the total variance, indicated temporal variation in virome composition: the May 2025 and February 2025 samples occupied relatively distinct positions, whereas the July, September, and December samples overlapped partially (Figure 2A). NMDS based on Bray–Curtis dissimilarity (stress = 0.109) showed similar partial temporal differentiation (Figure 2B). Hierarchical clustering further indicated that the two cage-associated samples (V and NV) collected in the same period tended to cluster closely, whereas the samples collected outside the cage area (M and O200m) clustered more variably (Figure 2C). Rarefaction curves showed variation in detected viral species richness across samples (Figure S1). PERMANOVA on the same Bray–Curtis dissimilarities indicated that sampling date accounted for 58.5% of the variation in virome composition (F = 3.17, *p* = 0.001), with homogeneous multivariate dispersion among dates (betadisper, F = 2.49, *p* = 0.12), so the separation reflects differences in composition rather than in within-group variability. Both temporal and local spatial factors may therefore contribute to variation in seawater virome composition, although the effects of site and sampling date cannot be fully separated in the present design; seasonal differentiation was also less pronounced in the overall virome composition than in the microbiome patterns reported in our previous study [5]. The temporal variation in the virome coincided with substantial seasonal changes in seawater temperature, which ranged from 10.7 ± 0.4 °C in February 2025 to 27.4 ± 1.3 °C in September 2024. However, temperature fitted as a continuous variable was not significantly associated with virome composition (envfit r^2^ = 0.24, *p* = 0.21; PERMANOVA R^2^ = 0.10, *p* = 0.21), indicating that the turnover did not follow a simple monotonic response to temperature. Marine viral communities respond to environmental conditions as well as to the abundance and physiological status of their microbial hosts, and seasonal variation in coastal virus–host populations has been reported previously [8,25]. Other physicochemical variables that can shape coastal microbial and viral communities, such as salinity, inorganic nutrients, chlorophyll a, dissolved oxygen, and water exchange driven by tidal and wind-driven flow, were beyond the scope of the present dataset, and the temporal patterns described here are therefore interpreted as correlative. The observed virome patterns may thus reflect seasonal environmental changes together with shifts in microbial potential host communities and their associated viruses.

### 3.2. Temporal Shifts in Dominant Viral Taxa

**Figure 3:**
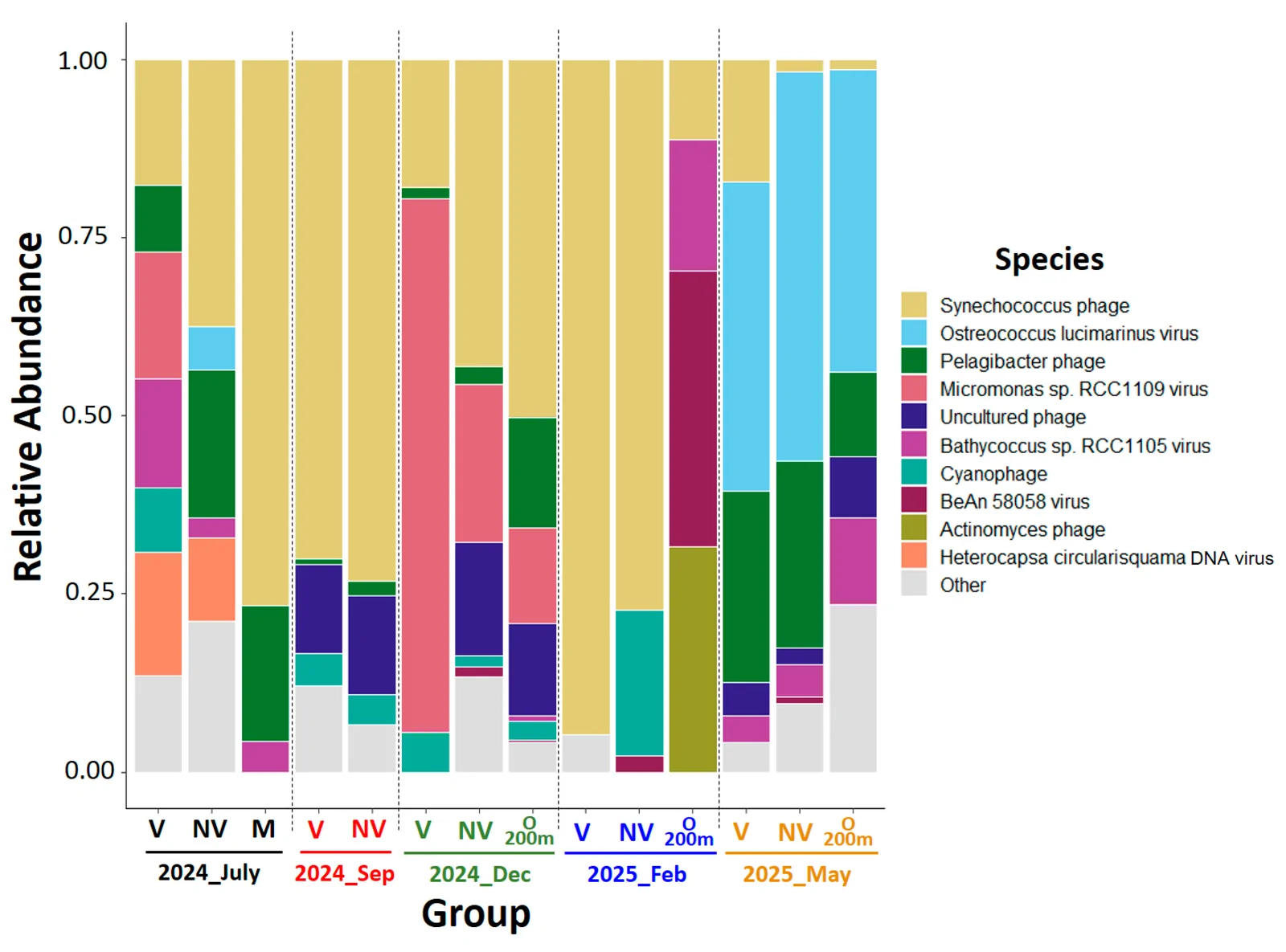
Relative abundance of the most abundant viral species at each sampling site and time point along the Geoje coastal area. Stacked bars show the relative abundance of the 10 most abundant viral species for each sample, expressed as the normalized viral coverage length ratio, that is, the percentage of aligned bases assigned to each species relative to the total aligned bases of all virus-like reads in the same sample. Bars are grouped by sampling date (2024_July, 2024_Sep, 2024_Dec, 2025_Feb, and 2025_May) and site (V, NV, M, O200m).

The seawater virome was dominated by bacteriophages and phytoplankton-infecting viruses and showed distinct temporal shifts in taxonomic composition (Figure 3). *Synechococcus* phages were major components of the virome across multiple sampling periods, and *Pelagibacter* phages and other cyanophages were also detected frequently. Viruses associated with picoeukaryotic phytoplankton showed particularly pronounced seasonal turnover: *Micromonas* sp. RCC1109 virus was prominent in December, whereas *Ostreococcus lucimarinus* virus and *Bathycoccus* sp. RCC1105 virus increased during the rewarming period in May. These temporal changes in dominant viral taxa were consistent with the seasonal structuring observed in the ordination analyses. However, not all database-based assignments were considered equally informative from an ecological perspective. For example, BeAn 58058 virus is a chordopoxvirus originally isolated from a rodent, whereas Actinomyces phages are not typically associated with coastal marine environments. Because taxonomic assignments were based on the best matches against a viral-only reference database, some reads may reflect limitations in database coverage or sequence similarities. These specific assignments are therefore reported for completeness but are not emphasized in the ecological interpretation. The succession of phytoplankton-associated viruses is ecologically relevant, because *Micromonas*, *Ostreococcus*, and *Bathycoccus* belong to the order Mamiellales and contribute importantly to marine picoeukaryotic primary production [26,27,28]. Viruses infecting these genera, including prasinoviruses, are widely distributed in marine environments and may regulate the turnover of their host populations [29,30]. The temporal replacement of dominant viral taxa may therefore be associated with seasonal changes in their potential hosts, and these virus–host dynamics may in turn influence marine carbon and nutrient cycling through virus-mediated host lysis and the subsequent release of cellular material into the surrounding water [8].

### 3.3. Associations Between Major Bacteriophages and Their Potential Host Populations

**Figure 4:**
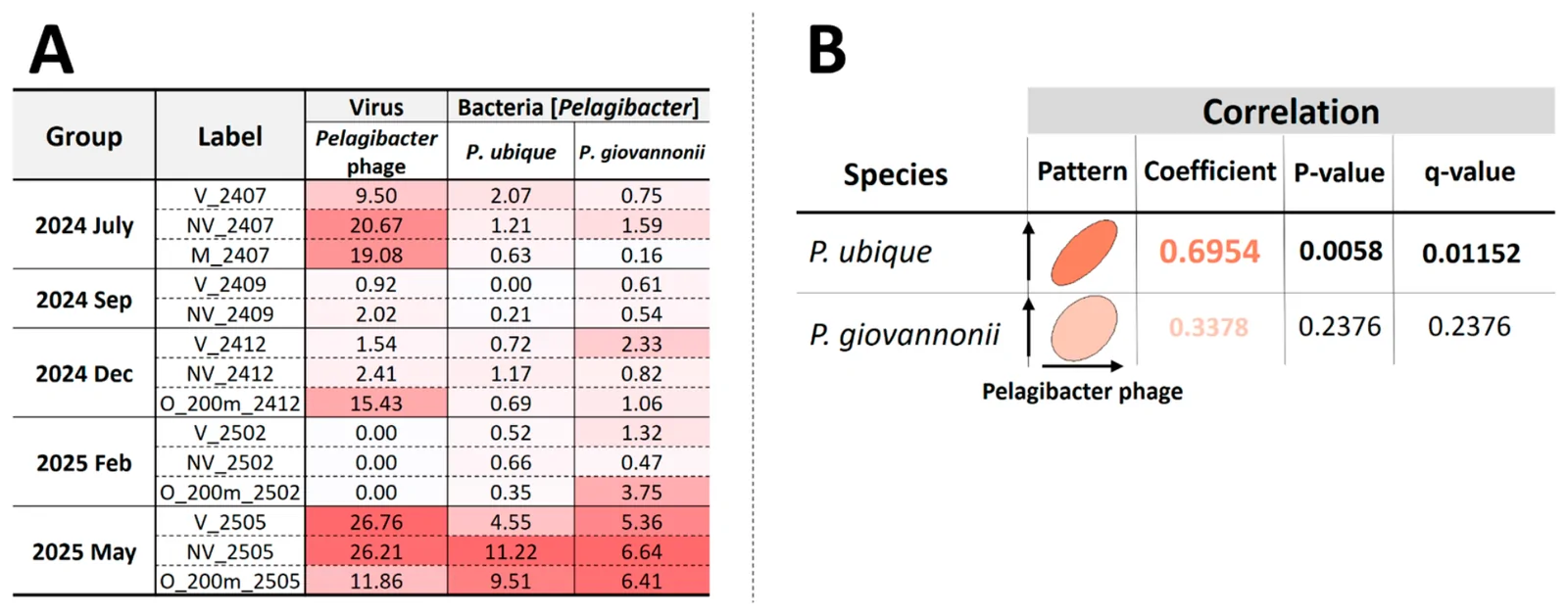
Abundance patterns and correlations between Pelagibacter phage and its potential bacterial hosts. (**A**) Normalized viral and prokaryotic coverage length ratios of Pelagibacter phage, P. ubique, and P. giovannonii across 14 samples collected during five sampling periods. Cell colors range from white to red with increasing abundance. (**B**) Pairwise Spearman correlations between Pelagibacter phage and each bacterial taxon, showing correlation patterns, Spearman’s correlation coefficients (ρ), unadjusted *p*-values, and Benjamini–Hochberg-adjusted *p*-values (q-values). Each correlation was calculated using all 14 samples, and *p*-values were adjusted across the two correlations shown. Associations with q < 0.05 were considered statistically supported within this exploratory analysis.

**Figure 5:**
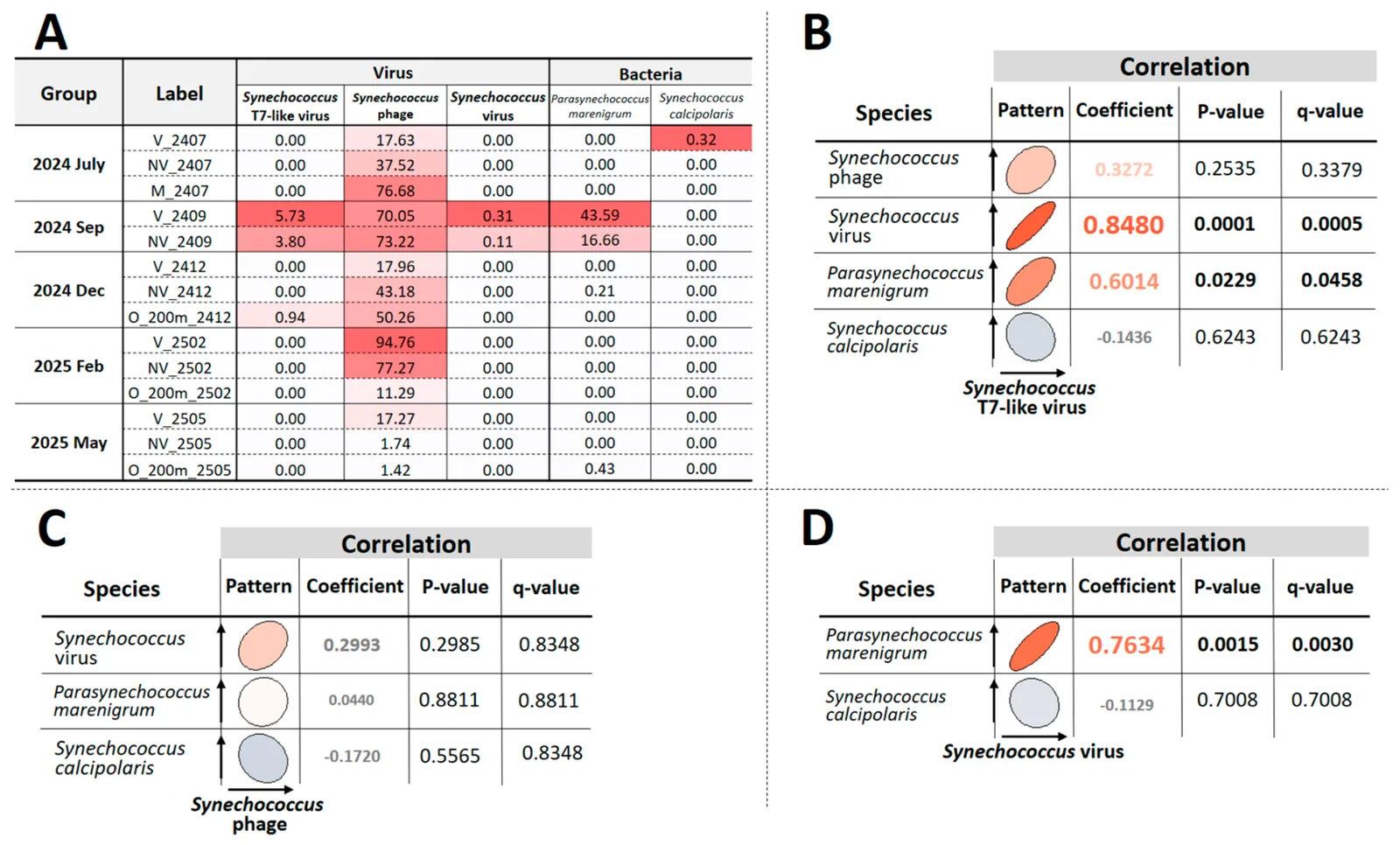
Abundance patterns and correlations among Synechococcus-associated viral and bacterial taxa. (**A**) Normalized viral and prokaryotic coverage length ratios for Synechococcus T7-like virus, Synechococcus phage, Synechococcus virus, Parasynechococcus marenigrum, and Synechococcus calcipolaris across 14 samples collected during five sampling periods. Cell colors range from white to red with increasing abundance. (**B**–**D**) Pairwise Spearman correlations of Synechococcus T7-like virus (**B**), Synechococcus phage (**C**), and Synechococcus virus (**D**) with the other indicated viral and bacterial taxa. Orange and blue ellipses indicate positive and negative correlations, respectively, and narrower ellipses represent stronger correlations. Spearman’s correlation coefficients (ρ), unadjusted *p*-values, and Benjamini–Hochberg-adjusted *p*-values (q-values) are shown. Each correlation was calculated using all 14 samples. Associations with q < 0.05 were considered statistically supported within this exploratory analysis.

To determine whether seasonal changes in the major bacteriophages were associated with variation in their potential bacterial hosts, the abundance patterns of *Pelagibacter*- and *Synechococcus*-associated viruses were compared with those of the related bacterial taxa (Figure 4 and Figure 5). *Pelagibacter* phage varied substantially over time (Figure 4A): abundance was relatively high in July 2024, fell markedly between September 2024 and February 2025, and rose again in May 2025, reaching normalized coverage length ratios of 26.76, 26.21, and 11.86 in the V, NV, and O200m samples, respectively. *Pelagibacter ubique* increased markedly over the same period, reaching values of 4.55, 11.22, and 9.51 in V, NV, and O200m, respectively. Correlation analysis supported a relationship between these populations: Pelagibacter phage abundance was positively correlated with that of P. ubique (ρ = 0.6954, *n* = 14, *p* = 0.0058, q = 0.0115), whereas its association with P. giovannonii was weaker and not statistically significant (ρ = 0.3378, *p* = 0.2376, q = 0.2376) (Figure 4B). This association is biologically plausible, because members of the SAR11 clade, including *Pelagibacter*, are among the most abundant heterotrophic bacteria in marine ecosystems and play a major role in dissolved organic carbon utilization [31,32], and pelagiphages infecting SAR11 bacteria are likewise widespread and abundant in marine viral communities [33]. Greater availability of potential hosts may increase encounters between phage particles and susceptible bacterial cells and thereby facilitate viral propagation, and the simultaneous increase in *P. ubique* and *Pelagibacter* phage observed in May is consistent with such host-associated phage dynamics. The seasonal variation in *Pelagibacter* phages may accordingly reflect changes in the abundance or community composition of their potential SAR11 hosts. The absence of a significant relationship with *P. giovannonii* nevertheless indicates that *Pelagibacter* phage abundance did not simply follow the total abundance of all *Pelagibacter*-related bacteria, which is consistent with a possible taxon-specific association. A correlation across 14 samples from a single annual cycle cannot, however, establish infection or host specificity: both populations may respond to shared seasonal drivers such as temperature, and the database-assigned “Pelagibacter phage” category may include several *pelagiphage* lineages with different host ranges.

More complex patterns emerged among the *Synechococcus*-associated viruses (Figure 5). *Synechococcus* phage was abundant across several sampling periods. In July 2024 its normalized abundance rose from 17.63 in V_2407 to 37.52 in NV_2407 and 76.68 in M_2407; high values persisted in September, at 70.05 in V_2409 and 73.22 in NV_2409; and the highest value was recorded in V_2502 in February 2025 (94.76). Abundance then fell markedly in May 2025. *Synechococcus* phage abundance therefore did not simply track seawater temperature, since high levels occurred under both relatively warm and relatively cold conditions. In the present study, individual *Synechococcus*-associated viral groups showed clearly different relationships with the bacterial taxa examined. *Synechococcus* T7-like virus was positively correlated with *Parasynechococcus marenigrum* (*p* = 0.0229, q = 0.0458) but not with *Synechococcus calcipolaris*, and *Synechococcus* virus was likewise positively correlated with *P. marenigrum* (*p* = 0.0015, q = 0.0030) but showed no significant relationship with *S. calcipolaris*. Both associations remained statistically supported after correction for multiple testing. Seasonal variation in *Synechococcus* and its associated viruses has also been observed in other coastal ecosystems: Sandaa and Larsen [25], for example, reported covariation between marine *Synechococcus* populations and their associated viruses over an annual cycle, together with seasonal replacement of viral populations, which emphasizes the importance of host availability and host-range differences in determining viral dynamics. Members of the genus *Parasynechococcus* are known to be highly abundant throughout the global ocean and to contribute substantially to marine primary production, so their high abundance here may indicate a substantial contribution of picocyanobacteria to local primary production [34]. An increase in *Parasynechococcus* abundance may in turn promote a subsequent increase in the associated bacteriophages by raising host availability. Temporal variation in bacteriophage populations may thus provide complementary information on host dynamics and on the environmental conditions of aquaculture waters. Notably, Synechococcus T7-like virus and Synechococcus virus were themselves strongly correlated (*p* = 0.0001, q = 0.0005), and their synchronous abundance patterns may reflect similar environmental responses, overlapping host populations, or common seasonal forcing. Both viral groups were detected primarily in September, when *P. marenigrum* also reached its highest abundance, which suggests that these populations may represent components of a temporally linked microbial assemblage. Because seawater temperature was also highest in September, these correlations may partly reflect a shared seasonal driver, and they are therefore best described as associations consistent with possible host relationships rather than as evidence of infection or host specificity.

### 3.4. Genotyping of the Pathogenic Megalocytivirus from Seawater Samples

**Figure 6:**
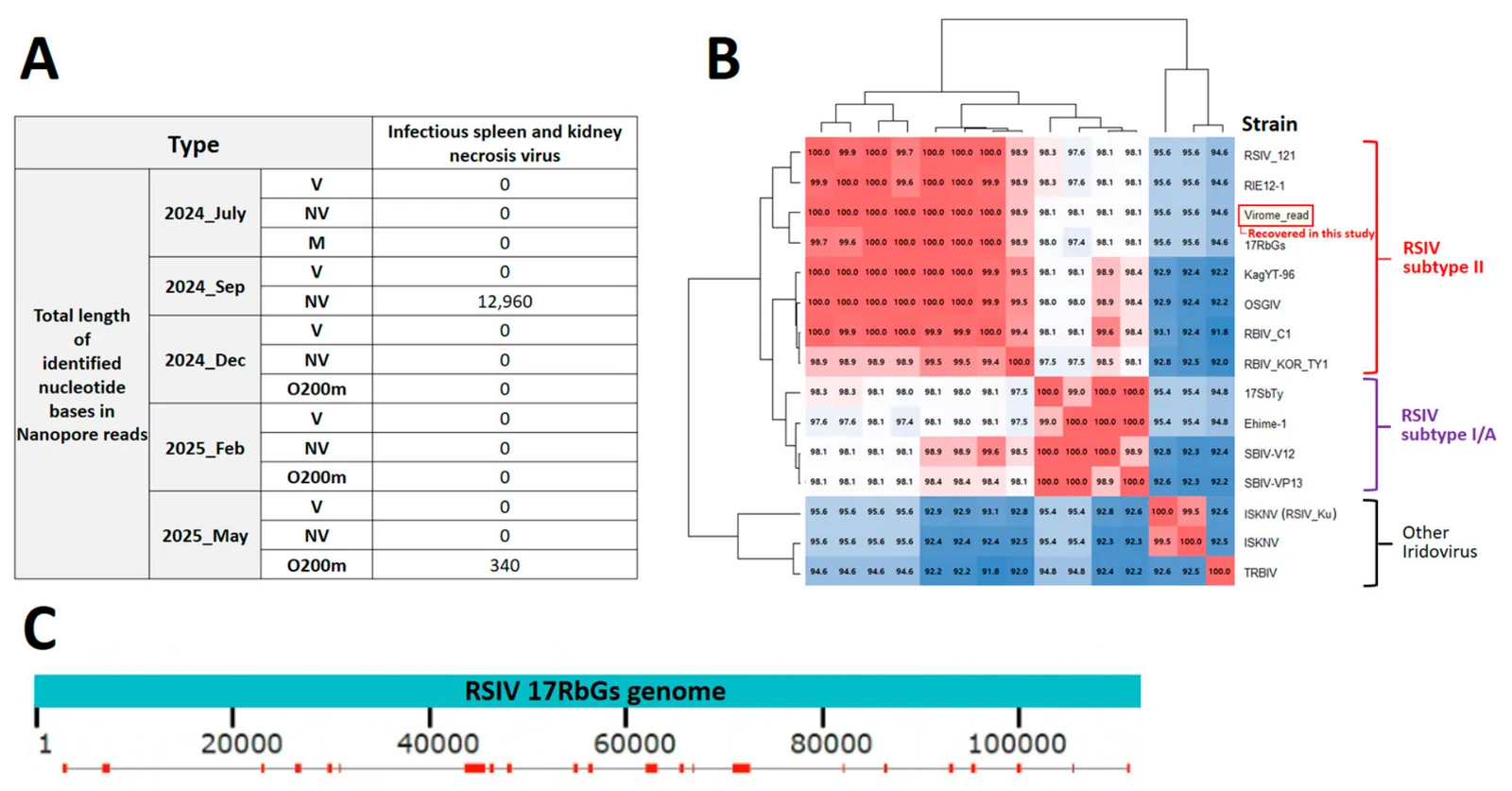
Metagenomic detection and sequence-based classification of RSIV-related sequences in the samples. (**A**) Total number of nucleotide bases in Nanopore reads assigned to infectious spleen and kidney necrosis virus (ISKNV)/red seabream iridovirus (RSIV; genus Megalocytivirus) in each sample. A total of 12,960 bases, derived from 25 non-contiguous reads, were recovered from the in-cage sample collected in September (2024_Sep_NV), whereas 340 bases were recovered from the offshore sample collected in May (2025_May_O200m). (**B**) Hierarchically clustered heatmap of pairwise average nucleotide identity (ANI) between the viral sequence recovered in this study (Virome_read) and reference iridovirus genomes. The heatmap color gradient represents ANI values, with red indicating higher ANI and blue indicating lower ANI. The recovered sequence clustered within the RSIV subtype II clade (red bracket), distinct from RSIV subtype I/A (purple bracket) and other iridoviruses (black bracket). (**C**) BLASTn alignment positions of the Nanopore reads on the RSIV 17RbGs reference genome. Red marks indicate where each read aligned; all alignments had bit scores ≥ 200. The reads mapped to discrete regions distributed across the genome rather than to a single contiguous segment.

Nanopore reads assigned to infectious spleen and kidney necrosis virus (ISKNV) on the basis of the NCBI Viral Reference Database were detected in samples collected in September 2024 and May 2025 (Figure 6A). The 12,960 bp recovered from the September 2024 sample (NV_2409) corresponded to 25 individual Nanopore reads that aligned to non-contiguous genomic regions. Each region was therefore supported by single-read coverage rather than a depth-based consensus. ISKNV belongs to the genus Megalocytivirus within the family Iridoviridae, and under the current ICTV taxonomy ISKNV, red seabream iridovirus (RSIV), and turbot reddish body iridovirus (TRBIV) are classified as distinct genotypes within the single species *Megalocytivirus pagrus 1*. Because several previous studies have nevertheless reported genetic differentiation among these three genotypes[11,12], homologous regions corresponding to the genomic intervals covered by the Nanopore reads were extracted from several genomes of ISKNV, RSIV, and TRBIV and subjected to ANI analysis in order to identify the genotype most closely related to the seawater-derived sequence. The Nanopore reads assigned to *Megalocytivirus pagrus 1* proved most closely related to the RSIV genotype and clustered specifically within RSIV subtype II. In a previous study, whole-genome ANI analysis classified RSIV strains into three distinct subtypes: I, II, and A [11], whereas the ANI values calculated here were based on the partial genomic regions recovered from seawater. Only partial genomic sequences of the seawater-derived RSIV were recovered, which provided insufficient resolution to distinguish subtype I from subtype A, but the virome-derived reads nonetheless showed high nucleotide similarity to RSIV subtype II and clustered consistently within the subtype II group. Previous studies have identified RSIV subtype II strains in rock bream and red seabream and have reported that rock bream is highly susceptible to this subtype [11,13]; consistent with those findings, the seawater-derived sequence detected at the rock bream cage farm was classified as RSIV subtype II. This finding is epidemiologically relevant, because RSIV subtype II has been reported as a predominant subtype in cultured marine fish in Korea [12,13], and complete-genome analysis of Korean isolates has identified RSIV subtype II in rock bream, the species cultured at the farm examined here [13]. Recovering RSIV-related sequences directly from seawater also suggests the potential of environmental virome sequencing as an environmental screening or early-warning tool in aquaculture. Previous studies have shown that RSIV DNA can be detected in seawater surrounding aquaculture facilities and that environmental DNA monitoring can reveal changes in viral abundance before the onset of observable disease; Kawato et al. [35], for example, detected RSIV environmental DNA at least five days before an RSIV disease outbreak in cultured fish, and iron flocculation has been shown to improve the sensitivity of RSIV detection from seawater substantially [36]. The recovery of RSIV-related sequences in the present virome dataset therefore suggests that metagenomic sequencing of concentrated seawater may complement targeted pathogen surveillance while simultaneously characterizing broader environmental viral communities. The detection of viral DNA in seawater alone does not, however, demonstrate active infection, viral replication, or disease in the farmed fish, the presence of infectious virus in the environment, or an outbreak at the farm; such DNA may originate from subclinically infected or recovered fish, from wild fish, or from non-infectious viral particles or free DNA transported by water currents. Confirmation of RSIV infection would require sampling of fish and validated diagnostic testing.

### 3.5. Limitations

This study has several limitations. First, without sub-0.22 μm fractionation, nuclease treatment, or viral-particle purification, the virome represents virus-associated sequences within total DNA, and the relative abundances of cell-associated and prophage-like sequences may differ from those in particle-enriched viromes. Second, the samples covered five time points within a single annual cycle and the non-cage sites differed between sampling periods, so seasonal effects cannot be fully separated from sampling-date effects and spatial and temporal effects are partly confounded; the observed patterns should therefore be read as those of the year investigated rather than as reproducible seasonal dynamics. Third, abundances were relative, based on a viral-only reference database, and derived from small numbers of virus-like reads in some samples. Fourth, the virus–bacterium correlations were exploratory. Finally, although RSIV-related sequences were successfully recovered from seawater, they were partial, did not cover the conventional marker genes, and were supported by single-read coverage, so the subtype affiliation should be regarded as provisional and their detection indicates the presence of viral DNA rather than infection or disease in farmed fish.

## 4. Conclusions

Long-read shotgun metagenomic sequencing of seawater concentrates revealed a dynamic DNA virome with statistically supported temporal structuring over one annual cycle in the coastal waters surrounding a rock bream sea-cage farm. The viral assemblages were dominated by bacteriophages associated with ecologically important marine bacteria, including *Synechococcus* and *Pelagibacter*, and by viruses infecting picoeukaryotic phytoplankton such as *Micromonas*, *Ostreococcus*, and *Bathycoccus*. The temporal succession of these viral groups, including the increase in *Micromonas*-associated viruses in December and the predominance of *Ostreococcus*-associated viruses in May, may be associated with seasonal turnover in their potential microbial hosts. Correlations between dominant bacteriophages and potential bacterial hosts further demonstrate the value of integrating viral and prokaryotic metagenomic data to understand microbial interactions in aquaculture-associated coastal environments. Alongside these ecological patterns, partial sequences of *Megalocytivirus pagrus 1* closely related to RSIV subtype II were identified, which highlights the additional potential of seawater virome analysis for environmental screening and early warning of aquatic viral pathogens. Seawater virome profiling thus provides a complementary and non-invasive approach for monitoring temporal microbial dynamics and detecting potential pathogen DNA signals in sea-cage aquaculture; such signals indicate the presence of pathogen DNA in the environment rather than infection or disease and require confirmation by fish sampling and validated diagnostic testing. Future studies incorporating RNA viruses, greater sequencing depth and temporal resolution, and validation against host-based clinical testing will be required to develop this approach further into an operational tool for ecological and biosecurity monitoring.

## Data Availability

The sequencing data analyzed in this study are available in the NCBI Sequence Read Archive (SRA) under accession number PRJNA1492777.

## Supplementary Materials

The following supporting information can be downloaded at: https://www.mdpi.com/article/10.3390/microorganisms14102362/s1, Figure S1. Rarefaction curves of viral species richness for each seawater sample. Curves show the number of viral species detected as a function of the number of virus-like sequences subsampled.

## Appendix A

**Table A1:** Accession numbers for the Nanopore sequencing data used in this study.

Group	Label	Accession
2024 July	V_2407	SRR39576957
	NV_2407	SRR39576956
	M_2407	SRR39576951
2024 Sep	V_2409	SRR39576950
	NV_2409	SRR39576949
2024 Dec	V_2412	SRR39576948
	NV_2412	SRR39576947
	O200m_2412	SRR39576946
2025 Feb	V_2502	SRR39576945
	NV_2502	SRR39576944
	O200m_2502	SRR39576955
2025 May	V_2505	SRR39576954
	NV_2505	SRR39576953
	O200m_2505	SRR39576952

**Table A2:** Total Nanopore sequencing output and the proportion of bases assigned by BLASTn to the NCBI Viral RefSeq database under different filtering criteria (E-value < 1 × 10^−5^; and E-value < 1 × 10^−5^ combined with ≥70% query coverage and ≥80% sequence identity).

Date	Label	Total Base	Estimated Virus Base [Ratio]	
			Ref_Viral DB *	Ref_Viral DB over Thresholds **
**2024_July**	V_2407	66.27 Mb	0.063 M [0.09%]	0.010 M [0.02%]
	NV_2407	52.56 Mb	0.062 M [0.12%]	0.010 M [0.02%]
	M_2407	16.96 Mb	0.024 M [0.14%]	0.009 M [0.05%]
**2024_Sep**	V_2409	42.45 Mb	0.716 M [1.69%]	0.264 M [0.62%]
	NV_2409	91.62 Mb	1.650 M [1.80%]	0.628 M [0.69%]
**2024_Dec**	V_2412	8.01 Mb	0.041 M [0.51%]	0.013 M [0.17%]
	NV_2412	47.8 Mb	0.196 M [0.41%]	0.037 M [0.08%]
	O_200m_2412	61.39 Mb	0.325 M [0.53%]	0.128 M [0.21%]
**2025_Feb**	V_2502	8.73 Mb	0.016 M [0.19%]	0.006 M [0.06%]
	NV_2502	17.64 Mb	0.065 M [0.37%]	0.012 M [0.07%]
	O_200m_2502	8.68 Mb	0.030 M [0.35%]	0.003 M [0.03%]
**2025_May**	V_2505	53.13 Mb	0.070 M [0.13%]	0.023 M [0.04%]
	NV_2505	50.07 Mb	0.086 M [0.17%]	0.031 M [0.06%]
	O_200m_2505	225.47 Mb	0.258 M [0.11%]	0.095 M [0.04%]

***** Sequence reads that matched entries in the NCBI Viral RefSeq database with an E-value < 1 × 10^−5^. ****** Sequence reads that matched entries in the NCBI Viral RefSeq database with an E-value < 1 × 10^−5^, query coverage ≥ 70%, and percent identity ≥ 80%.
