# Supplementary material for: Long-Read Metagenomics Reveals Temporal DNA Virome Dynamics and Recovers Sequences of a Major Viral Pathogen at a Sea-Cage Fish Farm
Source: Microorganisms. 2026 Oct 15;14(10):2362. doi: 10.3390/microorganisms14102362 (PMC13707698; doi:10.3390/microorganisms14102362)
Supplement: Supplementary file 1 [file microorganisms-14-02362-s001.zip › microorganisms-4562313-supplementary.pdf]

Supplementary Figure

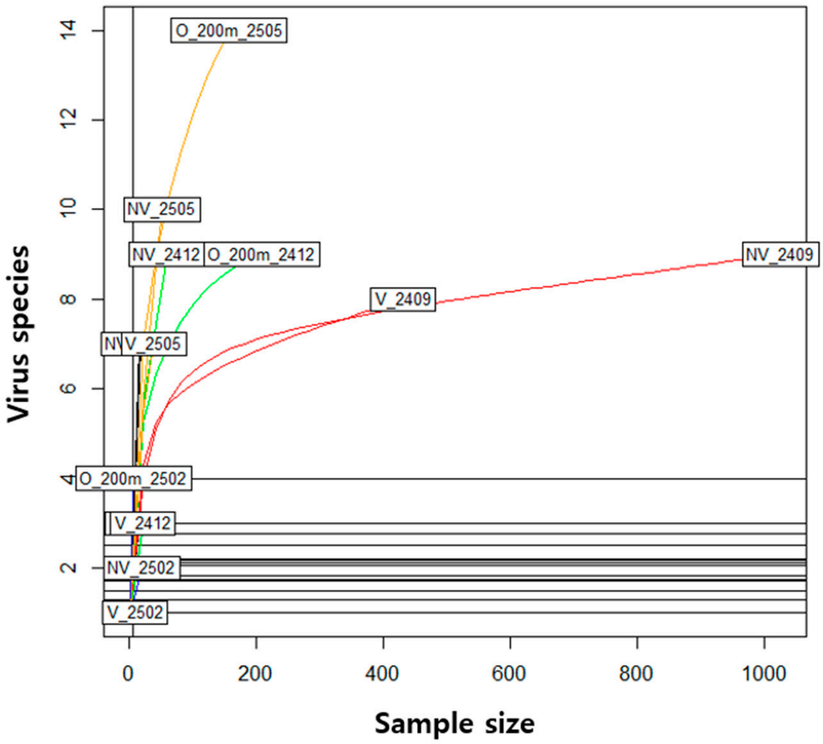

Figure S1. Rarefaction curves of viral species richness for each seawater sample. Curves show the number of viral species detected as a function of the number of virus-like sequences subsampled.
